# Optimization of the Hockey Fans in Training (Hockey FIT) weight loss and healthy lifestyle program for male hockey fans

**DOI:** 10.1186/s12889-017-4926-z

**Published:** 2017-11-28

**Authors:** Wendy Blunt, Dawn P. Gill, Shannon L. Sibbald, Brendan Riggin, Roseanne W. Pulford, Ryan Scott, Karen Danylchuk, Cindy M. Gray, Sally Wyke, Christopher Bunn, Robert J. Petrella

**Affiliations:** 1 Department of Family Medicine, Centre for Studies in Family Medicine, Schulich School of Medicine and Dentistry, Western University, London, Ontario Canada; 2 Lawson Health Research Institute, London, Ontario Canada; 3 School of Health Studies, Faculty of Health Sciences, Western University, London, Ontario Canada; 4 Graduate Program in Health & Rehabilitation Sciences, Faculty of Health Sciences, Western University, London, Ontario Canada; 5 The Schulich Interfaculty Program in Public Health, Schulich School of Medicine and Dentistry, London, Ontario Canada; 6 School of Kinesiology, Faculty of Health Sciences, Western University, London, Ontario Canada; 7 Institute of Health and Wellbeing, College of Social Sciences, University of Glasgow, Glasgow, United Kingdom; 8 Centre for Studies in Family Medicine, Western Centre for Public Health and Family Medicine, 2nd Floor, Western University, 1465 Richmond St, London, ON N6G 2M1 Canada

**Keywords:** Men’s health, Overweight/obesity, Lifestyle intervention, Weight loss, Process evaluation, sports fans

## Abstract

**Background:**

The health outcomes of men continue to be poorer than women globally. Challenges in addressing this problem include difficulties engaging men in weight loss programs as they tend to view these programs as contrary to the masculine narrative of independence and self-reliance. Researchers have been turning towards sports fans to engage men in health promotion programs as sports fans are typically male, and tend to have poor health habits.

**Methods:**

Developed from the highly successful gender-sensitized Football Fans in Training program, Hockey Fans in Training (Hockey FIT) recruited 80 male hockey fans of the London Knights and Sarnia Sting who were overweight or obese into a weekly, 90-minute classroom education and group exercise program held over 12 weeks; a 40-week minimally-supported phase followed. A process evaluation of the Hockey FIT program was completed alongside a pragmatic randomized controlled trial and outcome evaluation in order to fully explore the acceptability of the Hockey FIT program from the perspectives of coaches delivering and participants engaged in the program. Data sources included attendance records, participant focus groups, coach interviews, assessment of fidelity (program observations and post-session coach reflections), and 12-month participant interviews.

**Results:**

Coaches enjoyed delivering the program and found it simple to deliver. Men valued being among others of similar body shape and similar weight loss goals, and found the knowledge they gained through the program helped them to make and maintain health behaviour changes. Suggested improvements include having more hockey-related information and activities, greater flexibility with timing of program delivery, and greater promotion of technology support tools.

**Conclusions:**

We confirmed Hockey FIT was an acceptable “gender-sensitized” health promotion program for male hockey fans who were overweight or obese. Minor changes were required for optimization, which will be evaluated in a future definitive trial.

**Trial registration:**

NCT02396524 (Clinicaltrials.gov). Date of registration: Feb 26, 2015.

## Background

In 2014, 62% of Canadian men self-reported being overweight or obese versus 46% of women [1]. Excess weight is a significant contributor to the rise in many preventable chronic diseases [2] and premature mortality [3]. Up to 70% of men’s risk for these diseases could be prevented through a healthy diet, being physically active, smoking cessation, and reducing excess alcohol consumption [4, 5].

Men are continually underrepresented and underserved in health promotion interventions [6, 7]. Men are more likely to view weight loss programs as “feminized” domains, contrary to the male narrative of independence and self-reliance [8, 9]. Successful interventions targeting men’s health behaviours have been tailored to work with the masculine narrative rather than against it [10, 11]. Targeting male sports fans in professional sports club/team settings has shown recent success [12, 13] as these fans tend to share a group identity with other fans of the same sport [14] and have poor health habits [15, 16].

Two thirds of Canadians, predominantly middle-aged men, identify as hockey fans [17]. The Hockey Fans in Training (Hockey FIT) program capitalized on the large male fan base of major junior hockey in Canada to recruit men into a gender-sensitized weight loss and healthy lifestyle program. Hockey FIT was adapted from the successful Football Fans in Training (FFIT) weight loss program [12, 18] and integrated new components including the Health*e*Steps™ lifestyle prescription program [19] and an *e*Health online social network. Adaptation of the Hockey FIT program to Canadian hockey culture has been discussed in detail elsewhere [20]. An outcome evaluation of the Hockey FIT pilot program showed promising results with men in the intervention group losing, on average, 3.58 kg more than the comparator, and maintaining this weight loss to 12 months [21]. In this paper, we report on the process evaluation of a pilot pragmatic randomized controlled trial (pRCT) of the Hockey FIT program.

## Methods

### Aim

The aim of this process evaluation was to evaluate the acceptability of the Hockey FIT program by exploring the: 1) coaches' experience delivering Hockey FIT; 2) men’s experience with Hockey FIT; and 3) ways of optimizing Hockey FIT.

The Hockey FIT program and pRCT have been described in detail elsewhere [20]. Briefly, we recruited 80 male fans of two Ontario Hockey League (OHL) teams (London Knights and Sarnia Sting), aged 35–65 years, with a Body Mass Index (BMI) ≥28 kg/m^2^, and meeting the physical activity safety requirements. Men were randomized (1:1) to intervention (Hockey FIT program) or comparator (Wait-list Control, beginning Hockey FIT 12 weeks later).

The Hockey FIT program consisted of a 12-week active phase delivered at local GoodLife Fitness clubs or OHL team hockey arenas (when available), followed by a 40-week minimally-supported phase. The active phase involved 12 weekly, 90-minute classroom education and group-based exercise sessions delivered by trained Hockey FIT coaches. Coaches were graduate students and hockey team staff. In-between program sessions, participants were encouraged to engage in an incremental pedometer-based walking program and set/tracked lifestyle prescriptions for physical activity (steps), exercise and healthy eating. During the 40-week minimally-supported phase, participants were encouraged to access the following support tools: 1) the Health*e*Steps™ smartphone app providing tools to track and sustain physical activity; 2) a private online Hockey FIT social network (powered by Tyze Personal Networks: http://tyze.com/) for each site, only accessible to coaches and participants to share resources and support; 3) six motivational email messages (see Table 1) and an invitation to a 9 month booster session and reunion.

**Table 1:** Standardized Coach Messages Sent During 40 Week Minimally Supported Phase

Maintenance Week 4: How’s it going?
Hi Guys, Just wanted to take some time to check in and see how everyone is doing. I really enjoyed working with you guys during our 12 weeks together, and I hope that you have had success continuing to apply the healthy living knowledge you gained during the program. In addition, hopefully you all have managed to maintain your weight loss after the program ended; remember to continue to weigh yourself often to keep track of where you’re at. How have you guys been tracking your daily activities? The HealtheSteps™ app or on paper? Recall our discussion on barriers - it is important to plan for setbacks and use SMART goals if you find yourself going off track. It seems like you guys have been enjoying our online group thus far, please continue to use the group to post any healthy living, hockey, or just plain funny content. Hope everyone is well, Keep your stick on the ice.
Maintenance Week 11: Still going well?
Hi Guys, It’s now been some time since we finished the program, and hopefully everyone has been working hard to maintain their improvements. If you have not already done so, go back and check your average daily step count from at the end of the program. How does your current step count average compare? If the two averages are similar, what has helped you maintain your step count? Post some advice or tips that have helped you on the Dressing Room wall for the guys who haven’t been able to maintain their numbers. The HealtheSteps™ app is a great way of staying motivated and keeping track of your steps. Additionally, if you ever want to get in touch with the other guys to organize a ball hockey game, trip to see the Knights/Sting, or any other meet-up, feel free to use the messaging tool. Hope everyone is well, Keep your stick on the ice.
Maintenance Week 22: Still on target?
Hey Guys, Hope everything is well. Has everyone been able to maintain their end-of-program weight? Remember to weigh yourself weekly to track how much you have lost since the beginning of the program. If you have been losing weight, that’s awesome. As you now know, reducing your weight also reduces your risk for a variety of diseases, including heart failure, diabetes, and cancer. If your eating habits have been going a little off track, remember to make your goals SMART to keep focused. Just as a reminder, we will be organizing a little 9-month reunion so you will be able to see all the other guys and talk about how the months after the program have been going. Until then, continue to use the HealtheSteps™ App and post on the group to share recipes, exercises, articles, or other hockey stuff. Hope everyone is well, Keep your stick on the ice.
Maintenance Week 28: Looking forward
Hey Guys, It was great to see some of you at the reunion a few weeks ago. Even though we don’t have any more formal reunions planned for you guys before your final measurement session with us, we want to remind you to continue using the network to support one another and set up your own get-togethers. Speaking with some of you a few weeks ago, it sounds like you have organized a couple of exercise sessions on your own which is great to hear! If you haven’t met up with any of the guys but you would like to, don’t hesitate to post your ideas on your network and we are sure you will find some takers. Also, we would like to remind you once again to continue your daily or weekly weigh-ins to track your progress and if you find that you have reached a plateau or had a set-back, it may be time to go back to first principles and set some SMART goals again! Don’t get discouraged if you find yourself off-track, reach out to others on the network and I am sure that there will be loads of supportive words and ideas for you. Hope everyone is well, Keep your stick on the ice.
Maintenance Week 32: Reflecting on the process
Hey Guys, I hope you are starting to enjoy the warmer weather. I just wanted to check in and remind you to reflect on whether your exercise and diet routines are still healthy and that you are maintaining the changes that you had made during the 12 weeks of training sessions. You all did a great job incorporating your new knowledge about healthy lifestyles into your own daily living and we want to remind you how important it is to continue to use those skills. If you are ever questioning whether you have slipped back into old habits, set some SMART goals for next week and then reflect on whether you were able to meet them. If you find yourself running into new situations where making healthy choices may be difficult, ask others for suggestions about how they may have handled them (eg. Eating well while traveling, or exercising when at a cottage for the weekend). Keep your stick on the ice.
Maintenance Week 37: Hockey FIT for life?
Hey Guys, Congrats everyone, it has now been almost a year since you started the program. Now we want you to think back to those weight loss tips which you found most useful during the program and consider how often you still use them. Some people have found this program to be life-changing and we hope that you will continue to use the tools and tips that you learned as part of the program to maintain your weight loss or increased physical activity. We also want to take this opportunity to wish you good luck in the future and remind you to continue to use the HealtheSteps™ App and to stay in touch with each other using this network. You all supported each other so much during the program and maintaining those relationships or even reaching out to new friends will help you maintain your healthy lifestyle. Remember the network is also a great place to post ideas about pick-up hockey games or other activities that you may be getting involved in. I look forward to seeing you guys in a couple weeks and hearing about your successes in the past 12 months. Keep your stick on the ice.

Process evaluation data were collected from the intervention group (*n* = 40) alongside the pRCT at baseline, 12 weeks, and 12 months. Mixed methods were used to conduct the process evaluation using the following data sources: attendance records; coach interviews (CI) at the end of the active phase; focus groups (FG) with participants who completed the active phase; program fidelity (post-session coach reflections and program observations) collected during the active phase; and 12-month participant interviews (PI) during follow-up assessments after the minimally-supported phase. Convenience sampling was used to recruit participants for the focus groups at both sites, and for the 12-month participant interviews. All ‘program completers’ (*n* = 33), defined as those who attended ≥50% of active phase sessions including at least one session in the final 6 weeks, were invited to attend a focus group at their site. All ‘program completers’ who attended the follow-up at 12 months (*n* = 30) were invited to stay for an interview with a member of the research team. Program fidelity was monitored by two trained assessors (one main and one back-up), who observed all 12 sessions at both sites, and tracked the delivery of key tasks by the Hockey FIT coaches during each session, which includes whether they were delivered as designed in the program protocol [20] (see Table 2 for Weekly Topics and Table 3 for Program Observation Framework for Week 5). The main assessor completed post-session reflections with the head coaches after each session recording the coaches' experience of delivering key tasks at each session, and what the coach thought went well or did not go well.

**Table 2:** Weekly Topics

Topic	Key Tasks to Be Delivered	Fidelity Notes
Week 1: Introduction to Hockey FIT & Physical Activity Prescription	1. Introduction to Hockey FIT team & Men 2. Discuss program aims: “how to eat better, be more active and stay that way long term.” 3. Factors affecting our eating and activity 4. Energy balance (intake vs. output) 5. Introduction to Lifestyle Rx and Goal-Setting (set Physical Activity (step count) prescription (Rx)) 6. Walking Tour of Arena (by arena personnel)	Introduction to the Hockey FIT team was missed at both sites. Walking tour was missed as arena personnel were not available at either site.
Week 2: Healthy Eating Overview & Healthy Eating Prescription	1. Reintroduce Hockey FIT Team 2. Review participant goals from last week 3. Explanation of the food groups and eating a healthy diet 4. Formal introduction to S.M.A.R.T. goal setting 5. Receive Healthy Eating Rx and in groups set goals	Men were not split into groups to discuss healthy eating prescription in London.
Week 3: Meal Planning & Weight Loss	1. Review participant goals 2. Avoiding compensation 3. Example of individualized healthy eating plans 4. Health benefits associated with 5–10% long-term weight loss 5. Personal weight loss targets 6. Importance of support from others (including technology supports)	Men were not reminded of virtual support tools in London.
Week 4: Becoming Fit & Exercise Prescription	1. Review participant goals 2. Facts about exercise/becoming more active (including taking heart rate and rate of perceived exertion) 3. Overcoming barriers to exercise 4. Exercise Rx and setting goals 5. Local amenities	All tasks delivered at both sites.
Week 5: Alcohol & Weight Gain	1. Review participant goals 2. Alcohol and weight gain 3. Alcohol units 4. Planning your drinking 5. Cutting down on sugary drinks (fizzy and tea/coffee)	All tasks delivered at both sites.
Week 6: Stages of Change	1. Review participant goals (including alcohol) 2. Stages of Change 3. Introduction to setbacks and strategies for dealing with the men	Alcohol goals were not reviewed at both sites.
Week 7: Weight Loss	1. Review participant goals 2. Representation of weight loss achieved 3. Reflection on how things are going so far	All tasks delivered at both sites.
Week 8: Food Labels	1. Review participant goals 2. Understanding food labels and healthier foods 3. Importance of regular meals and breakfast	All tasks delivered at both sites.
Week 9: Eating Out	1. Review participant goals 2. Making favourite meals healthier 3. Eating out sensibly 4. Damage limitation for takeout	All tasks delivered at both sites.
Week 10: Avoiding Setbacks & New Exercise Prescription	1. Review participant goals 2. Common ideas about healthy living 3. Triggers for setbacks and how to avoid them 4. Set new Exercise Rx	All tasks delivered at both sites.
Week 11: Energy Balance & New Healthy Eating Prescription	1. Review participant goals 2. Set new Healthy Eating Rx 3. The energy balance and eating plans revisited 4. Locus of control revisited 5. Tour of dressing room	All arena sessions for the Sarnia group were held in the team players’ lounge (similar to the team dressing room), therefore there was no need to tour the dressing room during this session.
Week 12: Celebrating Achievements & Next Steps	1. Review of progress and next steps 2. Technology options	Technology options were not reviewed in London.

**Table 3:** Program Observation Framework – Session 5

Hockey FIT Session Five - Alcohol & Weight Gain	
Date, time, and venue	
# of participants present	
Classroom setup	
1. Welcome and Review Participant Goals – 10 min	
□ Take attendance and ask the men about adverse events □ Discuss problems achieving healthy lifestyle goals and strategies to overcome these difficulties □ Discuss current goals. □ Ask about use of local resources suggested in previous session. □ Update goal setting forms □ Remind men to use Healthy Living Tracking Form to track step counts, exercise minutes and heart rate, and healthy eating.	
2. Alcohol & Weight Gain – 10 min	
□ Understanding the part alcohol plays in weight gain can help the men start to consider ways to reduce or modify their alcohol consumption. If any of the men do not drink, speak to them about how pop and other high-sugar drinks can play a role in weight gain and how to reduce consumption. □ Complete activity on interesting facts about alcohol, ask about calories in alcohol, writing down findings on flip chart for all to see. □ Ask for suggestions about why alcohol makes you gain weight and write them down. □ Lead a discussion on Interesting Facts about Alcohol Sheet to illustrate that alcohol leads to weight gain in many ways.	
3. Myths About Alcohol & Alcohol Units – 15 min	
*Exploring the myths about alcohol can encourage the men to think about their own drinking habits* *Content, Style & Method of Delivery* □ Have men complete the Alcohol Quiz □ After the quiz, discuss with the men each of the answers and keep score. □ Make a point about what a “drink” is with visual supports □ Discuss with men the link between alcohol and chronic disease □ Remind the men current recommendations suggest 2 alcohol free days per week	
4. Planning your Drinking – 15 min	
*Alcohol is an enjoyable part of socializing and does not need to be cut out completely. The next exercise encourages men to make educated choices about drinking.* *Content, Style & Method of Delivery* □ Remind the men how alcohol consumption can affect weight loss as in order to lose weight they need to burn off more calories than they take in. □ Suggest that changing their drinking habits could really help them lose weight. □ Ask men for ideas about how to enjoy a drink without increasing their beer belly. Write the suggestions on the flip chart. □ Lead a discussion on barriers around cutting down drinking (including on game days). How could they be overcome? 5. Cutting Down on Sugary Drinks – 7 min □ Remind the men to be careful about non-alcoholic drinks too as many fizzy drinks are also high in calories. Suggest the men drink much more water or at least the diet pop options. □ Remind the men they should also be careful about what they put in coffee and tea as one tablespoon of sugar adds nearly 50 cal and they should try to use semi-skimmed (or, better still, skimmed) milk instead of cream. □ Suggest the men set a drinking-related goal	
Active Session – 40 min	
*Content, Style & Method of Delivery* □ Warm up and provide 30 min of aerobic workout followed by a cool down. □ Cater for different levels of fitness. □ Remind the men to work at their own level by monitoring their RPE and Heart Rate	

Fifteen participants (5 in London, 10 in Sarnia) from the intervention group attended the 12-week focus groups (one focus group was held in London and another in Sarnia); all four coaches (a head coach and an assistant coach in each club) completed 12-week interviews; and 28 participants (14 in London, 14 in Sarnia) from the intervention group completed 12-month interviews. Ethical permission was granted by the Western University Health Sciences Research Ethics Board and all participants and coaches provided written informed consent.

Coach and focus group transcripts were analyzed together with three members of the research team, one an expert in qualitative research, and guided by the framework approach [22]. An inductive approach was used to code the data line-by-line. The research team met and a framework of overarching codes was developed off of which new codes could be added based on the findings from the 12 month interviews. The 12-month interviews were reviewed by the research team and new codes were added to the framework. Fidelity data (program observations and post-session coach reflections) were read through by two members of the research team and summarized. Key tasks missed were identified through the program observations and supplemented by the post-session coach reflections along with suggestions for improving program delivery. The fidelity data was then ‘triangulated’ [23] with the findings from the focus groups, and interviews with coaches and participants, and additional codes were added to the framework. The final framework with all data sources was used to summarize the findings for publication.

## Results

**Fig. 1:**
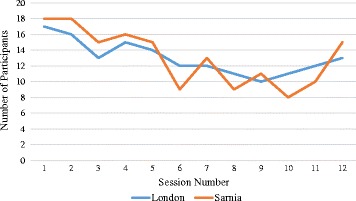
Weekly Attendance (Intervention group, separated by site)

Based on the fidelity data, of the 51 tasks to be delivered by the coaches over the 12 weeks, 46 tasks were delivered (84% completion) in London and 49 tasks were delivered (96% completion) in Sarnia (See Table 2). Attendance did drop mid-program at both sites (see Fig. 1), but increased again during the final program sessions.

### Coaches' experience delivering Hockey FIT

Coach interviews and post-session coach reflections informed the coaches' experience delivering Hockey FIT. Coaches enjoyed seeing the men progress throughout the program and the friendships that were developed, *“…to see these guys from day one, they were nervous, coming in to a baseline assessment and not knowing exactly what they were doing, and then from week 12 where now they’re trying to organize golf games together and really enjoying each other’s company, so that was fun to watch. And then from a research side it was also extremely fun because you could physically see changes in these guys”* (CI-2).

The coaches valued the relationships they developed with participants knowing they were helping the men improve their lifestyles, “*My favorite part was just interacting with these guys every week and really building a rapport with them and knowing within myself I’m helping people, I’m helping 15 guys out here that want to be helped”* (CI-1).

Some coaches felt they were “lecturing” participants at the beginning of the program in part due to the greater amount of classroom content and becoming familiar with the flow of the sessions. This changed as the program progressed, *“Once we got into sessions 4 and 5 though I thought that it started to flow, it was a lot easier to deliver, it wasn’t quite as much me lecturing, it was more sort of facilitating discussions among them…”* (CI-4). One of the coaches also identified feeling more comfortable with the physical activity portion of the program compared to delivering the nutrition information, *“I felt more comfortable doing the exercise [components]. I’ve been coaching since I was 15 so it’s easy for me now that I can try to motivate people and I have a better idea of the body in a physical aspect than I do in a nutritional aspect”* (C1–1)*.*

As indicated by Figure 1, the attendance dropped mid-program. A coach described how they felt this made the program more difficult to deliver, *“that was a barrier to overcome because a lot of the program is interaction and you want guys to be talking and coming up with ideas so if you only have 9 in a group and you only have one group of 5, one group of 4, it’s a lot harder than if you had 4 groups of 4 or 5, where you have more ideas coming out”* (CI-1).

The coaches did have suggestions for improving the program. One coach expressed concern about participants exercising on the concrete surface of the arena as this can exacerbate previous injuries, *“…I didn’t feel like I was giving them the exercises that they needed and again that was because we didn’t really take into account the environment that we’d be doing those exercises in.”* (CI-4). Timing was a common difficulty noted by coaches throughout the post-session reflections and interviews, *“I wish it was longer, not only number of weeks but I wish it was a 2 hour program instead of an hour and a half because sometimes they had a lot more questions than we anticipated”* (CI-3). More hockey-related content was also suggested by coaches to take greater advantage of the men’s common interest, *“…if we did more [hockey] drills with balls and sticks and things like that because they were all such big hockey fans…maybe incorporating more of what they all had in common, which was hockey, would have been nice”* (CI-2).

### Men’s experience with Hockey FIT

Participants in the intervention group were on average, 49.1 (SD 9.1) years of age, with the majority being married or common law (87.5%), of Caucasian ethnicity (95%), and having greater than a high school education (67.5%). At baseline, the average weight of participants was 112.5 (SD 24.6) kg, with a BMI of 36 (SD 5.9) kg/m^2^ and an average step count of 6859.6 (SD 3253.8) steps per day. Further information on the baseline characteristics of participants has been discussed elsewhere [21].

#### Active phase

Men’s experience with the active phase of the Hockey FIT program was explored through focus groups. Men joined Hockey FIT for a variety of reasons including weight loss, increasing physical activity levels, and a desire to improve their health for their families, *“I want to live to see my family and my kids grow up and get married. I lost my dad at 13 and he was 33… it would be nice to, I passed him, but go way past him. Double.”* (FG-1).

Most men appreciated how other members of the group were similar in size, shape, and desire to lose weight and improve their lifestyle, *“we all have the general understanding that we’re all in the same boat”* (FG-2). Participants compared themselves to each other and were quick to point out those who they felt did not belong, *“Some of it was we wanted to improve our self-image and we didn’t like being compared to the model on the front of the magazine concept, and yet we had two [models] in the class”* (FG-1). Others noted that the variation of exercise activities supported the range of physical abilities present in the group, *“I liked the variation because I am not as fit as some of the other guys here, I used to be, but I’m not…”* (FG-2).

The classroom content was valued by participants as this information both reinforced and added new knowledge to help participants make lifestyle changes, *“…understanding exercise and target heart rate, its impact on obviously your fitness or your calorie burn, those are the things I didn’t know”* (FG-1). One of the men explained how this new knowledge led to actual behaviour changes, *“I never ate breakfast for 51 years, and now I haven’t missed breakfast in probably six weeks”* (FG-1).

Participants felt the coaches were invested in their success in the program, *“They really had a genuine interest in health and well-being and us”* (FG-2). The men felt accountable to show up for the program and their coaches as they considered themselves a team, “*It’s that whole teamwork…you’ve got to show up for the team”* (FG-2). The men’s experience with the program left them eager to attend the next session, *“The combination of everything, I wanted to learn, the next week I wanted to see what else. And the competition and everything. Everybody said they just wanted to come back”* (FG-2).

Some participants felt the program was rushed to cover all of the content, *“I found that with the class and the exercise it was almost you know, 3 hours squeezed into an hour and a half”* (FG-1). Others were disappointed with the lack of hockey-related content and support from the OHL team personnel, *“I thought that maybe the [OHL] trainer would come out and talk to us a bit about the training, and health, and sports, and exercise”* (FG-2). Some participants suggested involving a dietitian or a chef in a session, *“I can only cook so many types of food, so why not have a chef come in and go over different things to show us how to do it properly”* (FG-1). Lastly, some of the men would have enjoyed competition between sites, *“It would have been nice to see the competition between the Sarnia Sting and the London Knights”* (FG-2).

#### Minimally-supported phase

Interviews conducted with the men at 12 months informed the participants experience maintaining their healthy lifestyle during the minimally-supported phase. Participants found the skills they had gained through the active phase of the program helped them to maintain their health behaviour changes, *“…the lessons I learned during Hockey Fit continued to follow me, in the sense of the water, the fruits and the vegetables”* (PI-3, Site 1). The emphasis on making simple lifestyle changes helped participants continue to be physically active, *“I think the attention to the simple thing as walking and staying somewhat fit has helped me stay focused on maintaining what I learned in the program and keep it going”* (PI-7, Site 1). Others noted the skills they learned through the handbook was helpful in maintaining their weight loss goals, *“it was plain and simple, it was on paper, this is what you should do and if you follow this you’re probably going to lose weight”* (PI-2, Site 2). The pedometer was also cited as helpful for maintaining physical activity levels after the program was completed, *“…even the pedometer, it’s a really good thing because every day I was very cognizant of that thing and I always made sure that I was watching it…”* (PI-14, Site 2).

Participants found they were encountering each other at community events, hockey games, and arenas and found it helpful to talk about their progress, *“I was out in [rural city], out in nowhere at a hockey arena and just walking and walking and then I ran into one of our guys, he was playing with the other team and so it was funny, and are you getting your steps in, so it was just part of the program…”* (PI-2, Site 1).

Men indicated the fear of regressing back into old habits as a reason for continuing with their healthy lifestyle changes, “*I’ve seen these results, but I don’t want to regress”* (PI-4, Site 1). Participants did identify barriers in maintaining their healthy lifestyles including medical conditions, injuries, and difficulties being active in the winter, “*I would say the biggest challenge that I faced is winter. I think it’s tough to move in the winter and that tends to pack on weight.”* (PI-14, Site 1).

#### Technology support

The Health*e*Steps™ smartphone app was introduced to the men during session 11. It was not used by participants due to technical challenges experienced by the men (i.e., difficulties signing in, not tracking steps, crashing). Based on the 12 month interviews, the Hockey FIT social network was used passively with men only accessing the network when they received a message/post from their coach or another participant. Men admitted their own and other participants lack of interaction on the social network limited the potential of the network to support their progress during the minimally-supported phase, *“It might help if more people were active on it, but I wasn’t active on it either so I can’t complain about it. I’m just saying if there’s more interaction going on than it might have inspired more people,”* (PI-7, Site 2).

Nevertheless, many men found the standardized messages (see Table 1) sent regularly from their coaches through the social network (and also via email) helpful as they reminded participants about the importance of maintaining their healthy lifestyles, *“They don’t shame you into doing it but they give you a good reminder of what you’re doing, makes you think about what you’re not doing”* (PI-12, Site 2). Some participants would have valued more personal messages from the coaches as they felt left behind once the program ended.

#### 9 Month Booster Session/Reunion

Eight out of 40 participants from the intervention group (4 out of 20 from London, 4 out of 20 from Sarnia) attended the booster session/reunion. Participants were asked at the 12 month interview why they could not attend. Reasons participants did not attend included work (*n* = 5), family commitments (*n* = 3), too far to travel (*n* = 4), not aware of the event/what it entailed (*n* = 2), or other commitments (*n* = 7). Participants noted this event was held on Super Bowl weekend, during the winter, and only in London making it difficult for some to attend.

The eight who attended enjoyed seeing each other again and found it highly motivating to hear about others’ success, *“It’s kind of neat to see the guys, a couple of the guys did really well so it was kind of cool to see, it makes you think that you can actually accomplish something”* (PI-2, Site 2). Some of the men found this session/reunion set them back on track with their goals, *“It actually brought me back from a couple of weeks of falling off the wagon if you can call it that. Really because of the timing of it, it was towards the middle of winter, it really helped to actually say okay, yea you can still find some ways to do it…”* (PI-9, Site 1)*.*

### Optimizing Hockey FIT

Based on the interviews with coaches and participants, a number of areas were identified for optimizing the program for future delivery. These items included improving mid-program attendance, coach training, nutrition education, timing, exercise modifications, amount of hockey skills and drills, app usability, the booster session/reunion, and the Hockey FIT social network (See Table 4). Overall, these items were minor and would not require significant changes to the program design.

**Table 4:** Items for Optimization

Item	Description	Optimization
Mid-Program Attendance	There was a drop in attendance at both sites mid-way through the program.	The latter half of the program will be run in the arena with special guests in attendance to increase attendance during these sessions.
Coach Training	Coaches noted being less confident delivering the classroom components, particularly at the beginning of the program.	eLearning modules for coaches are being developed to allow for remote training of coaches and for easier access to training materials throughout program delivery. Coaches will be required to complete and pass a test in order to be a certified Hockey FIT coach.
Nutrition Education	Coaches found they were less confident delivering the nutrition components as the men would have questions they could not always answer.	A dietitian has been added to the research team to assist with training coaches on nutrition and to provide resources and tools to assist the coaches with educating participants in nutrition. A handout on junk food (trans fats, saturated fats, and cholesterol) has been added to compare to the healthier options.
Timing	Coaches found the timing was difficult to follow as some content elicited more conversation within the group than others.	Key tasks for program delivery have been outlined in the coaches handbook for each session to ensure coaches are aware of what tasks must be covered in each session. There are also optional activities included to fill in time if needed.
Exercise Modifications	Coaches felt they needed more exercise options to better meet the group needs particularly at the start of the program.	Modifications have been added to the coach handbook for the exercise components to better meet the variety of participant abilities (Week 1).
Hockey Skills and Drills	Coaches identified that participants were disappointed with the lack of hockey skills and drills throughout the program.	More hockey related facts and exercises will be included through the program. More sessions at the arena in the latter half of the program will increase the connection to hockey.
App Usability	Technical difficulties with the Health*e*Steps™ smartphone app prevented participants from using the app.	The technical glitches with the app have been resolved with the latest updates. Coaches will be trained on how to use the app within the program (i.e., heart rate monitoring during exercise), and
Booster Session and Reunion	This event was only held in London, and was on the weekend of the Super Bowl resulting in some participants not attending due to distance and conflicting events.	Having a booster session and reunion in both London and Sarnia and allowing both London and Sarnia participants to attend one of these sessions at either sites would provide more options for more men to attend.
Hockey FIT Social Network	Participants were not highly engaged in the use of the social network, only checking the network if a post by a coach or another participant was made.	A Hockey FIT Social Network smartphone app is being developed. Coaches will post different tasks each week during the 40 week minimally-supported phase for both the intervention and wait-list groups. Participants will receive goals (points) for their team for completing the tasks and posting these on the Hockey FIT Social Network or updating their coach via e-mail if they do not have regular computer access. This includes posting healthy recipes, activities, weekly achievements, etc. The team with the most points at the end of the maintenance phase wins the “Memorial Fan Cup” and an extra prize.

## Discussion

Findings from this process evaluation demonstrate Hockey FIT is highly acceptable – to both participants and coaches – for promoting healthy lifestyles and weight loss in male hockey fans who are overweight or obese. Previous research surrounding men’s health interventions have emphasized the importance of tailoring program materials to the male narrative [11]. Results from this process evaluation confirm the importance of tailoring interventions to the male narrative and supports the opportunity to further improve men’s health through engaging sports fans. Hockey FIT participants appreciated the program recruited men of similar interest regarding hockey, body size, and weight loss goals. Men felt a connection to each other, and in turn felt obligated to attend sessions and make lifestyle changes in order to contribute to their team. Suggested improvements included adding more hockey related skills and drills, sessions at the arena, more in-depth coach training and nutrition education, exercise modifications, greater opportunity for participants to attend the booster session, and more pro-active use of the Hockey FIT social network to better promote long-term support for health behaviour changes made by the men during the program.

Many of the qualitative findings from the focus groups mirrored the quantitative results of the program exit questionnaires completed by the men directly after the active phase of the program. These quantitative results were published in detail elsewhere [24]. According to results from the questionnaire completed at 12 weeks, 96% of participants found the exercise and educational classroom sessions beneficial to making health behaviour changes [24]. During the focus groups, men had expressed excitement and enthusiasm for coming back to the next session to learn more. All 27 Hockey FIT participants who completed this questionnaire indicated they had made changes to their eating habits as a result of being in the program [24]. In the focus groups, there were men who indicated they were now eating breakfast regularly because of Hockey FIT. Although the quantitative results from this questionnaire were positive and important in supporting the acceptability of the program, the focus groups and interviews provided a richer context over which these behaviour changes occurred. This allowed the research team to further understand why certain program components worked well or not well, how Hockey FIT impacted the men’s lives both in the short and long term, and the ways in which Hockey FIT can be improved to better meet the needs of participants.

Findings from Hockey FIT including a desire for more sport-specific activities were similar to findings from the FFIT process evaluation [25]. The Hockey FIT coaches also enjoyed delivering the program and meeting participants, but felt pressured to stay on time similarly to FFIT [25]. We found participants had a strong desire to compete between sites, which speaks to the longstanding rivalry between the two participating major junior hockey teams [26]. In contrast to FFIT, the standardized messages sent during the 40 week minimally-supported phase were viewed more favourably by the Hockey FIT participants as the men in Hockey FIT may have valued more information and support from coaches to help maintain their health behaviour changes. We also saw a drop in attendance mid-program which may have been a result of running the program in the summer (i.e., vacation).

### Study limitations

There were several limitations to this study. The two head coaches may have responded more positively towards the program during the interview as they were both heavily involved in the development of the Hockey FIT program and materials. Only 15 participants (10 in Sarnia, 5 in London) out of 30 invited participants attended the focus groups limiting the generalizability of these findings to the wider group. Moderators noted one of the focus group participants was more vocal about his experiences with the program than others in the group. This may have swayed the results of the focus group more heavily in favour of this participants’ experience rather than the entire group. Data were only collected from the intervention group; collecting data from the wait-list group could have deepened our understanding of participants’ experience with the program. Lastly, there was a low turn-out for the booster session due to a variety of factors (i.e., Super Bowl, weather, and location) limiting the amount of data collected about this event.

## Conclusions

This study supports the findings of other gender-sensitized programs targeting sports fans [12] and provides an innovative and effective approach for engaging men in health promotion interventions and improving men’s health. Hockey FIT was found to be a highly acceptable program by both participants and coaches involved in the pilot. Our results indicate only minor changes are needed to optimize Hockey FIT for future implementation in a definitive trial.

## Abbreviations

BMI: Body mass index
CI: Coach interviews
FFIT: Football fans in training
FG: Focus groups
Hockey FIT: Hockey Fans in Training
OHL: Ontario Hockey League
PI: Participant interviews
pRCT: pragmatic Randomized Controlled Trial
SD: Standard deviation
